# HDAC1 inhibition by MS-275 in mesothelial cells limits cellular invasion and promotes MMT reversal

**DOI:** 10.1038/s41598-018-26319-2

**Published:** 2018-05-31

**Authors:** Lucia Rossi, Cecilia Battistelli, Valeria de Turris, Valeria Noce, Clemens Zwergel, Sergio Valente, Alessandra Moioli, Andrea Manzione, Marco Palladino, Veronica Bordoni, Alessandro Domenici, Paolo Menè, Antonello Mai, Marco Tripodi, Raffaele Strippoli

**Affiliations:** 1 Department of Cellular Biotechnologies and Hematology, Section of Molecular Genetics, Sapienza University of Rome, Rome, Italy; 2 Center for Life Nano Science@Sapienza, Istituto Italiano di Tecnologia, Rome, Italy; 3 Department of Chemistry and Technologies of Drugs, Sapienza University of Rome, Rome, Italy; 4 Department of Clinical and Molecular Medicine, Sapienza University of Rome, Nephrology Unit, Sant’Andrea University Hospital, Rome, Italy; 5 Gene Expression Laboratory, National Institute for Infectious Diseases “Lazzaro Spallanzani” I.R.C.C.S., Rome, Italy

## Abstract

Peritoneal fibrosis is a pathological alteration of the peritoneal membrane occurring in a variety of conditions including peritoneal dialysis (PD), post-surgery adhesions and peritoneal metastases. The acquisition of invasive and pro-fibrotic abilities by mesothelial cells (MCs) through induction of MMT, a cell-specific form of EMT, plays a main role in this process. Aim of this study was to evaluate possible effects of histone deacetylase (HDAC) inhibitors, key components of the epigenetic machinery, in counteracting MMT observed in MCs isolated from effluent of PD patients. HDAC inhibitors with different class/isoform selectivity have been used for pharmacological inhibition. While the effect of other inhibitors was limited to a partial E-cadherin re-expression, MS-275, a HDAC1-3 inhibitor, promoted: (i) downregulation of mesenchymal markers (MMP2, Col1A1, PAI-1, TGFβ1, TGFβRI) (ii) upregulation of epithelial markers (E-cadherin, Occludin), (iii) reacquisition of an epithelial-like morphology and (iv) marked reduction of cellular invasiveness. Results were confirmed by HDAC1 genetic silencing. Mechanistically, MS-275 causes: (i) increase of nuclear histone H3 acetylation (ii) rescue of the acetylation profile on E-cadherin promoter, (iii) Snail functional impairment. Overall, our study, pinpointing a role for HDAC1, revealed a new player in the regulation of peritoneal fibrosis, providing the rationale for future therapeutic opportunities.

## Introduction

The peritoneum is a serosal membrane that forms the lining of the abdominal cavity. Peritoneum is composed by a continuous monolayer of mesothelial cells (MCs), cells of mesodermal origin with an epithelial-like cobblestone shape. MCs cover a sub-mesothelial region formed by bundles of collagen fibers and other extracellular matrix (ECM) proteins with few fibroblasts, mast cells, macrophages, and vessels. MCs secrete mucins facilitating the movements between visceral and parietal layers^1^. Moreover, through production of factors active on coagulation, fibrinolysis, cytokines and chemokines, MCs regulate serosal homeostasis and leukocyte trafficking^2^.

Peritoneal fibrosis is a pathological process leading to progressive alteration of peritoneum morphology and functions. Peritoneal fibrosis has been observed in a variety of pathological conditions, including prolonged practice of peritoneal dialysis (PD), a renal replacement therapy for patients with kidney disease, post-surgery adhesions, peritoneal metastases^2,3^. Peritoneal inflammation and ensuing fibrosis remains a critical issue in the long-term outcome of PD, which is often hampered by altered permeability of the peritoneal membrane, as a result of infection or chemical stress. High osmolality solutions required for water ultrafiltration and convective drainage of waste products in the uremic milieu, are believed to play a direct role in phenotypic rearrangement of MCs upon few years of daily PD exchanges^4^. Occasional episodes of peritonitis may amplify this process, leading to the dramatic picture of encapsulating peritonitis or plain fibrosis, both settings that may force the patient into a premature switch to hemodialysis.

MCs have an important role in peritoneal fibrosis due to induction of epithelial to mesenchymal transition (EMT), characterized by acquisition of invasive features and secretion of profibrotic/proangiogenic mediators^5–7^. Due to their peculiar features, the transition of MCs has been recently characterized as a mesothelial to mesenchymal transition (MMT)^3^.

With regard to fibrosis occurring in PD patients, continual exposure to hyperosmotic, hyperglycemic, and acidic dialysis solutions, mechanical stress connected to dwelling practice, and episodes of catheter complications (including peritonitis and hemoperitoneum) may cause acute and chronic inflammation and injury of the peritoneal membrane, evolving in MMT and fibrosis. Among the wide array of extracellular factors implicated in this process, TGFβ1 proteins play a major role. In mice models of PD, the intraperitoneal injection of adenovirus carrying TGFβ1 gene induced a peritoneal fibrosis similar to that induced upon exposure to PD fluids^8^. On the other hand, TGFβ1 blocking peptides preserved the peritoneal membrane by PD fluid induced damage^9^.

*Ex vivo* analysis of MCs derived by effluent of PD patients shows that these cells maintain a mesenchymal-like state even after removal of fibrogenic stimuli^10–12^.

This stable acquisition of a new gene expression pattern suggests the involvement of epigenetic mechanisms. Thus, the main goal of this study is to analyse the role of epigenetic modifications occurring during the induction of MMT in MCs and to evaluate the potential of EMT reversal (mesenchymal to epithelial transition, MET) upon treatment with specific pharmacological inhibitors or genetic silencing. In particular, here we focused on the impact of histone acetylation.

Histone acetylation and deacetylation play an essential role in modifying chromatin structure and in regulating gene expression in eukaryotic cells^13,14^. Hyperacetylated histones are generally found in transcriptionally active genes, whereas hypoacetylated histones are associated to transcriptionally silent regions of the genome. Key enzymes, which modify histone proteins and thereby regulate gene expression, are histone acetyltransferases (HATs) and histone deacetylases (HDACs). In mammals, both these acetylating/deacetylating enzymes are components of multiprotein complexes containing other proteins known to exert their role in transcriptional activation/repression. To date, eighteen distinct human HDACs have been reported, grouped into four classes (I-IV) depending on their primary homology to *Saccharomyces cerevisiae* HDACs (RPD3, HDA1, and SIR2).

The implication of HDACs in EMT has been demonstrated by recent studies, especially in tumors. Due to the heterogeneity of experimental models analyzed, HDAC inhibition has been demonstrated both to promote and inhibit tumor EMT and invasiveness^15–18^.

In order to characterize HDACs specific role in the onset and maintenance of MMT and peritoneal fibrosis, and therefore to contribute to unveil potential pharmacological approaches to this pathological condition, we made use of MCs isolated from peritoneal effluent of PD patients and tested the effects of specific HDAC inhibitors.

We revealed a specific role for class I HDAC1-3, and in particular for HDAC1: its inhibition by treatment with MS-275 compound causes the reversal of MMT-related marker gene expression and the reacquisition of epithelial-like morphology; moreover, treated cells showed a marked reduction in motility. When focusing on mechanisms, we analysed in particular molecular bases of Snail functional inactivation by MS-275.

Overall, our study, focusing on a new regulator of MC MMT, may provide rationale for therapies aimed at counteracting peritoneal fibrosis.

## Results

### MCs derived from peritoneal effluent of PD patients undergo reversal of MMT-related marker gene expression when treated with MS-275, a selective HDAC1-3 inhibitor

To analyze the role of HDACs in the maintenance of a mesenchymal-like state in MCs that have undergone MMT *in vivo*, we used MCs isolated from peritoneal effluent of PD patients. When cultured *in vitro* these cells have been shown to maintain a stable cobblestone-like or a fibroblastic phenotype, and their stage of trans-differentiation correlates with the progression of peritoneal damage and with the time of PD treatment^19^. Even with a cobblestone-like phenotype, these cells are different from normal healthy MCs since they express lower levels of E-cadherin and cytokeratins 8–18, and express Snail^11,20^.

**Figure 1:**
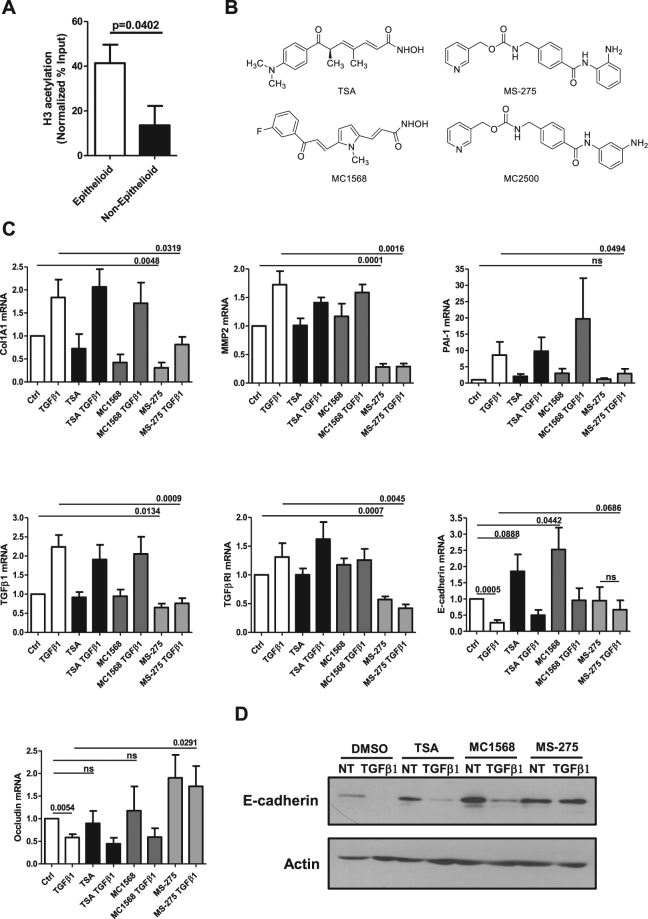
Effect of Class I and Class II HDAC inhibition on epithelial and mesenchymal markers expression in MCs derived from peritoneal effluent of PD patients. (**A**) qPCR analysis of ChIP assays with anti-acH3 antibody and, as controls, normal rabbit IgG on chromatin from epithelial-like (epithelioid) MCs (left) and mesenchymal-like (non-epithelioid) MCs (right). Data show the enrichment of H3 acetylation on human E-cadherin promoter. Values derived from three independent experiments are normalized respect to the IP efficiency (evaluated through GAPDH promoter amplification). Data are reported as means ± SEM and expressed as ((IP-IgG) %Input). (**B**) Chemical structures of HDAC inhibitors used in this study. (**C**) MCs were treated with DMSO vehicle (Ctrl) or with TSA (TSA) (30 nM), MC1568 (10 μM), MS-275 (250 nM) for 72 h; samples were also either left untreated or were treated with TGFβ1 (2 ng/ml) for the last 24 h. Expression of Col1A1, MMP2, PAI-1, TGFβ1, TGFβRI, E-cadherin and Occludin was evaluated on total RNA by qRT-PCR. Bars represent means ± SEM of 5 experiments (for Col1A1 and Occludin n = 4). p-values are reported in the graphs. (**D**) Representative Western blot experiment of 5 performed showing expression of E-cadherin from cell lysates of MCs treated as above. Actin was used as a loading control. P < 0.05 was considered significant.

To analyze whether histone deacetylation activity plays a role in epithelial gene expression changes during MMT, histone H3 acetylation on E-cadherin promoter was analyzed by chromatin immunoprecipitation (ChIP) in epithelial-like (epithelioid) and mesenchymal-like (non-epithelioid) MCs. The observation of higher H3 acetylation levels on E-cadherin promoter in epithelial-like with respect to mesenchymal-like cells suggests a role for HDAC activity in MMT induction during PD (Fig. 1A).

To demonstrate the effect of HDACs in the regulation of genes relevant for the maintenance of a mesenchymal phenotype, ECM production and invasiveness, MCs were treated for 3 days with Trichostatin A (TSA), a pan HDAC inhibitor; with MC1568, an HDAC4-6-8 inhibitor, and with MS-275, a HDAC1-3 inhibitor^21–23^ (Fig. 1B). While treatment with TSA and MC1568 was ineffective, MS-275 significantly decreased the expression of mesenchymal markers such as type I collagen (Col1A1), MMP2, and PAI-1 (Fig. 1C). Accordingly, the expression of both TGFβ1 and TGFβRI were decreased upon treatment with MS-275 (Fig. 1C). On the other hand, the expression of epithelial markers (E-cadherin and Occludin) was increased upon treatment with MS-275 (Fig. 1C). While TSA and MC1568 induced a partial recovery of E-cadherin expression, they failed to promote a more general reprogramming towards an epithelial-like phenotype.

**Figure 2:**
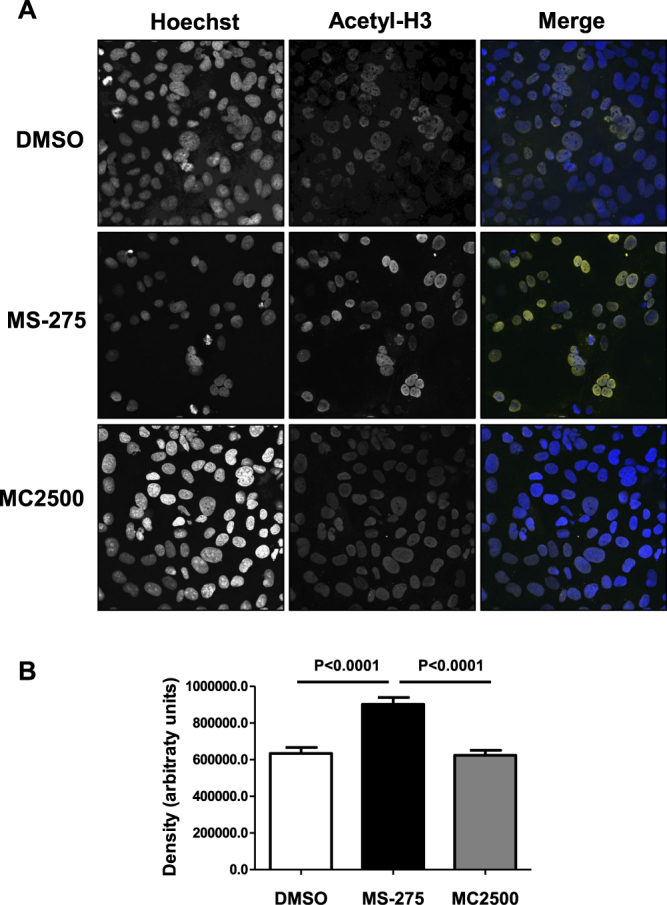
Treatment with MS-275 promotes nuclear histone H3 acetylation. (**A**) Confocal immunofluorescence analysis of MCs treated with DMSO, MS-275 (250 nM), MC-2500 (250 nM) for 12 h. Cells were fixed, permeabilized and stained with anti-acetylated histone H3 (yellow). Images were acquired by confocal microscopy. Nuclei were stained with Hoechst 33342 (blue). Data are representative of three independent experiments. (**B**) Quantification of the experiment shown in (A). The histogram shows fluorescence intensities of nuclear acetylated histone H3 staining quantified using the software ImageJ. Bars represent ± SEM. At least 400 nuclei were analyzed per condition. P < 0.05 was considered significant.

Interestingly, MCs treated with MS-275 were unresponsive to contemporary TGFβ1 stimulation. The increase of E-cadherin expression was confirmed at protein level (Fig. 1D). Increase of TSA concentrations up to 250 nM led to a partial rescue of epithelial, but not to a decrement of mesenchymal markers (Suppl. Fig. 1A–C). As a verification of MS-275 specificity, treatment with compound MC2500, an inactive *meta* isomer of MS-275, did not modify the expression of these epithelial/mesenchymal genes. (Suppl. Fig. 2A–D)^24^. In the same conditions, immunofluorescence (IF) analysis confirmed an increase of histone H3 acetylation in the nuclei of MCs treated with MS-275, but not with compound MC2500 (Fig. 2A,B). Since MS-275 is up to 13-fold more potent against HDAC1 than against HDAC2 and -3, and at the used dose (250 nM) it is specific towards HDAC1 inhibition, we checked the effect of genetic HDAC1 silencing in our model^21^.

**Figure 3:**
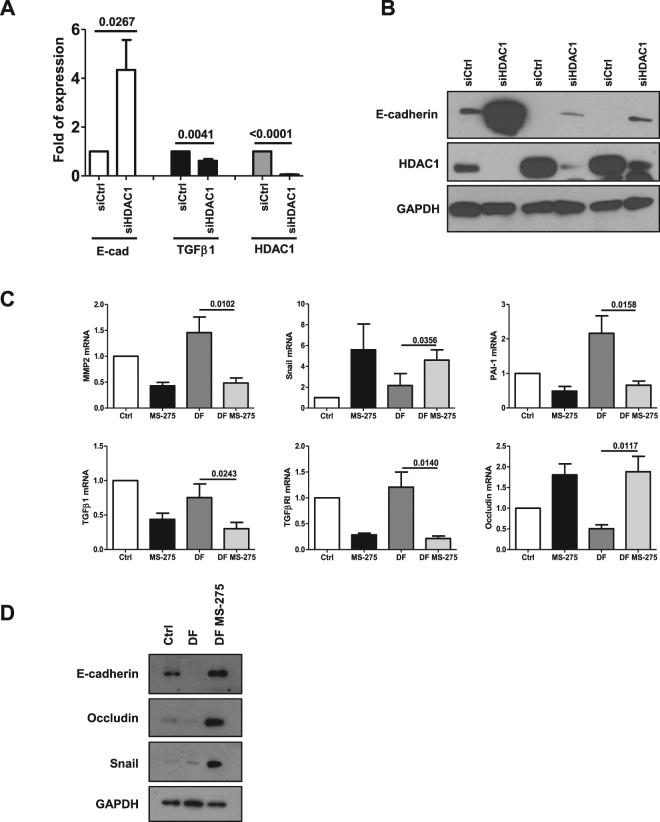
Effect of HDAC1 siRNA silencing on E-cadherin and TGFβ1 expression in MCs and effect of MS-275 in blocking MMT induced by exposure to PD fluid. (**A**) Quantitative RT-PCR was performed on total RNA from MCs transfected with either control (siCtrl) or specific HDAC1-targeting siRNAs (siHDAC1). Expression of E-cadherin, TGFβ1, HDAC1 was evaluated. Bars represent means ± SEM of 5 experiments. (**B**) Western blots showing the expression of E-cadherin and HDAC1 in total cell lysates of MCs from three different PD patients transfected with either control or specific HDAC1-targeting siRNAs. GAPDH expression was evaluated as a loading control (**C**) Effect of MS-275 on MMT induced by exposure to PD fluid. Quantitative RT-PCR was performed on total RNA from epithelial-like MCs treated for seven days with stay safe balance 4.25% (used at a concentration of 1:1 with complete 199 Medium). Alternatively, cells were treated for four days with stay safe balance 4.25% (used at the same concentration as before) and then treated with the same PD fluid in the presence of MS-275 for three more days. As a control, MCs were left untreated for four days and then treated with MS-275 or DMSO for the last three days. Expression of MMP2, Snail, PAI-1, TGFβ1, TGFβRI, Occludin was evaluated on total RNA by qRT-PCR. Bars represent means ± SEM of 4 experiments (**D**) Western blots showing the expression of E-cadherin, Occludin and Snail in total cell lysates of MCs treated as above. GAPDH expression was used as a loading control. Representative experiment of 4 performed. P < 0.05 was considered significant.

Notably, the specific HDAC1 targeting, by siRNA approach, shown in Fig. 3A,B, was found sufficient to induce E-cadherin and to markedly limit TGFβ1 expression. To directly analyze the effect of HDAC1 in mediating the changes induced by PD fluids, epithelial-like MCs were treated with stay safe balance PD fluid. The induced MMT was reversed by subsequent treatment with MS-275, as demonstrated by RT-PCR and WB analysis (Fig. 3C,D).

These results demonstrate that while the effect of TSA and MC1568 is limited to a partial rescue of E-cadherin expression, MS-275 treatment, targeting HDAC1 function, is causal for MMT reversal. Moreover, MS-275 was able to restore an epithelial signature after MMT induction upon exposure *in vitro* to PD fluids.

### Treatment with MS-275 mediates the reacquisition of an epithelial-like phenotype and the inhibition of cellular motility and invasion

The effect of MS-275 has been analyzed also at morphological level by IF analysis.

**Figure 4:**
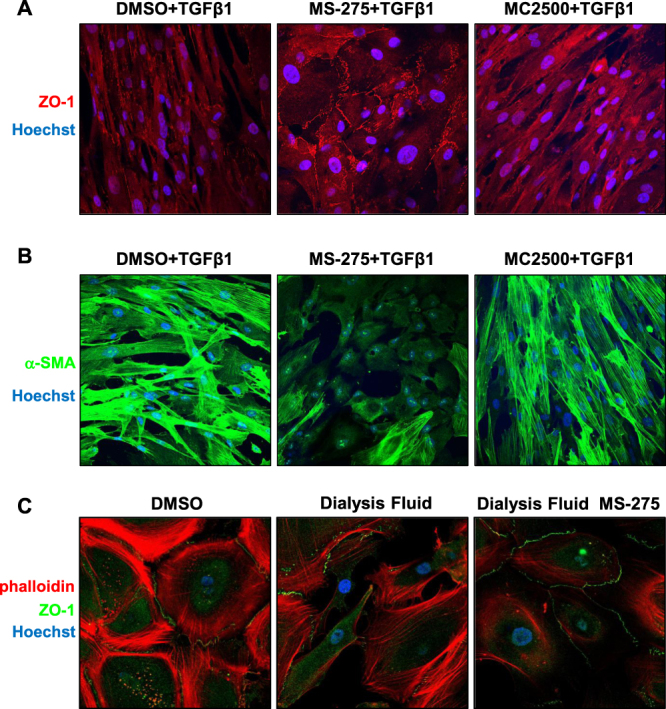
MS-275 promotes ZO-1 plasma membrane localization, α-SMA downregulation and the reversal to an epithelial-like morphology independently of treatment with TGFβ1. Effect of MS-275 treatment on ZO-1 and α-SMA expression and localization. (**A**) MCs were treated with MS-275 (250 nM), MC2500 (250 nM) or DMSO for 72 hours. Moreover, cells were left untreated or stimulated with TGFβ1 for additional 24 hours. Only TGFβ1-treated cells are shown. Cells were fixed, permeabilized and stained with a polyclonal antibody against ZO-1. Images were acquired by confocal microscopy. Cell nuclei are shown in blue (Hoechst 33342). Confocal images are shown from one representative experiment of three performed. (**B**) MCs were treated as in (**A**). Cells were stained with a monoclonal antibody against α-SMA. Confocal images are shown from one representative experiment of three performed. (**C**) Effect of MS-275 on MMT induced by exposure to PD fluid. Epithelial-like MCs treated for four days with stay safe balance 4.25% and then treated with the same PD fluid in the presence of MS-275 for three more days. Alternatively, MCs were treated with stay safe balance 4.25% for seven days or left untreated for four days and then treated with DMSO for the last three days. Cells were fixed, permeabilized and stained with phalloidin and with a polyclonal antibody against ZO-1. Images were acquired by confocal microscopy. Cell nuclei are shown in blue (Hoechst 33342). Confocal images are shown from one representative experiment of three performed. Overlay images are shown. Single fluorescences are shown in Suppl. Fig. 6. P < 0.05 was considered significant.

Treatment with MS-275 induced the recovery of fibroblastic MCs towards a cobblestone-like cell shape, which was associated to a marked relocalization of ZO-1 at cellular junctions (Fig. 4A and Suppl. Fig. 3A), and to a general decrease of α-SMA expression (Fig. 4B). Interestingly, MCs reacquiring a cobblestone phenotype were insensitive to contemporary TGFβ1 stimulation (Fig. 4A,B and Suppl. Fig. 3B,C). In accordance with molecular data, MS-275 blocked the induction of a fibroblastic-like shape and the loss of ZO-1 membrane localization in epithelial-like MCs treated with high glucose PD fluid (Fig. 4C and Suppl. Fig. 4).

During the process of peritoneal fibrosis, invasion of the sub-mesothelial stroma by MCs is a key event leading to abnormal production of ECM proteins and pathological angiogenesis^25^. Here, we analyzed the impact of MS-275 in MCs motility performing an *in vitro* scratch assay on confluent monolayers of MCs from PD patients.

**Figure 5:**
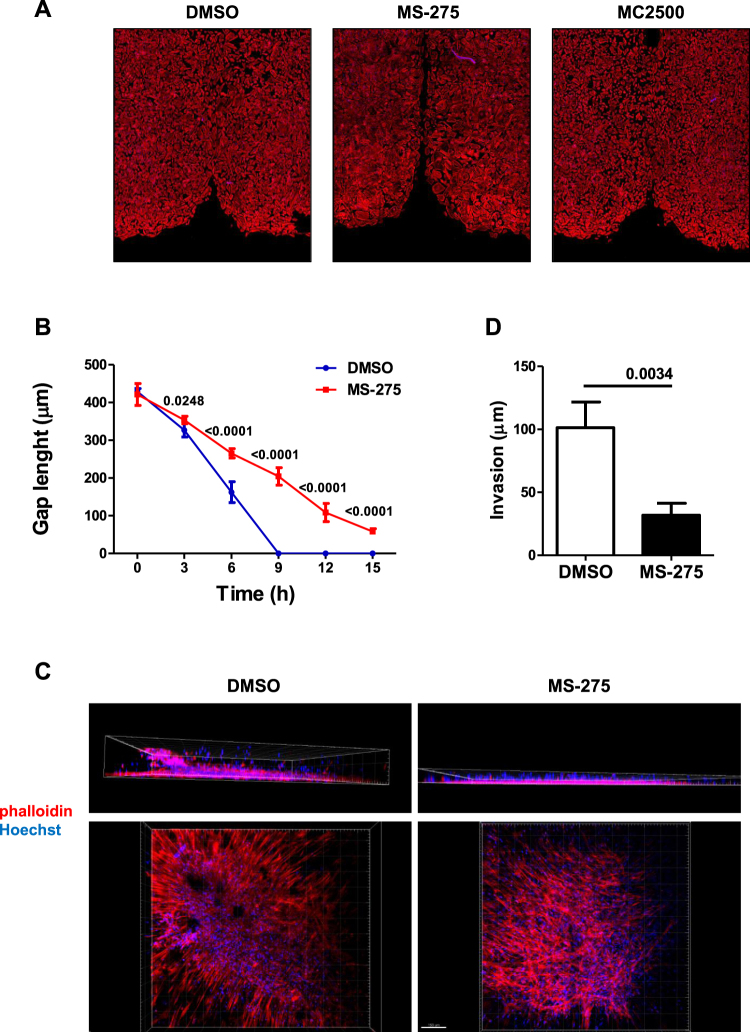
Treatment with MS-275 mediates inhibition of directed cellular motility as well as of three dimensional invasion. (**A**) Effect of treatment with MS-275 on wound closure. MCs from patients undergoing PD were allowed to reach 100% confluency in an Ibidi μ-Dish. MCs were pre-treated with DMSO, MS-275 (250 nM) or MC2500 (250 nM) for 48 h in culture medium supplemented with 10% FCS. Therefore, the insert was removed and after 18 h cells were fixed and stained with phalloidin (red) or Hoechst33342 (blue) to stain nuclei. Representative experiment is shown of three performed. (**B**) MCs were treated as above. After removal of the insert, the wound area was photographed every 30 min for 15 h by bright field microscopy. The width of the wound was measured and the wound closure rate was calculated. 9 different points from 3 different scratches were analyzed per condition. The result described is representative of 3 independent experiments. Values of MS-275-treated cells compared to DMSO-treated cells are reported. **(C**,**D**) Effect of treatment with MS-275 on three-dimensional invasion by MCs from patients undergoing PD. (**C**) MCs were pretreated (24 h) with DMSO or MS-275 (250 nM) and then overlaid with a Matrigel matrix. Invasion was monitored over 48 h. Three-dimensional invasion was enhanced by adding 20% FCS to the well. Cells were fixed and stained with phalloidin (red), and Hoechst 33342 (cell nuclei; blue). Top: *xz* maximal projection; Bottom: *xy* acquisition at the top of the well. Scale bar: 100 μm. Representative experiment is shown, of 3 experiments performed in duplicate. (**D**) Quantification of the experiment shown in (**C**). Cellular invasion was quantified through Huygens (SVI) and visualized through Imaris image analysis software. P < 0.05 was considered significant.

Treatment of MCs with MS-275 led to a partial reduction of scratch closure, whereas treatment with MC2500 was ineffective (Fig. 5A,B). We then analyzed whether exposure to MS-275 may affect three-dimensional (3D) invasion through Matrigel. We found that treatment with MS-275 totally abolished MCs invasion (Fig. 5C,D).

These results demonstrate that treatment with MS-275 promotes reversal towards an epithelial-like morphology of MCs having undergone MMT *in vivo*, strongly limits MCs directed migration while totally abolishing 3D invasion, thus impacting important features of the MMT/fibrotic program.

### Treatment with MS-275, while inducing Snail expression, hampers its activity

**Figure 6:**
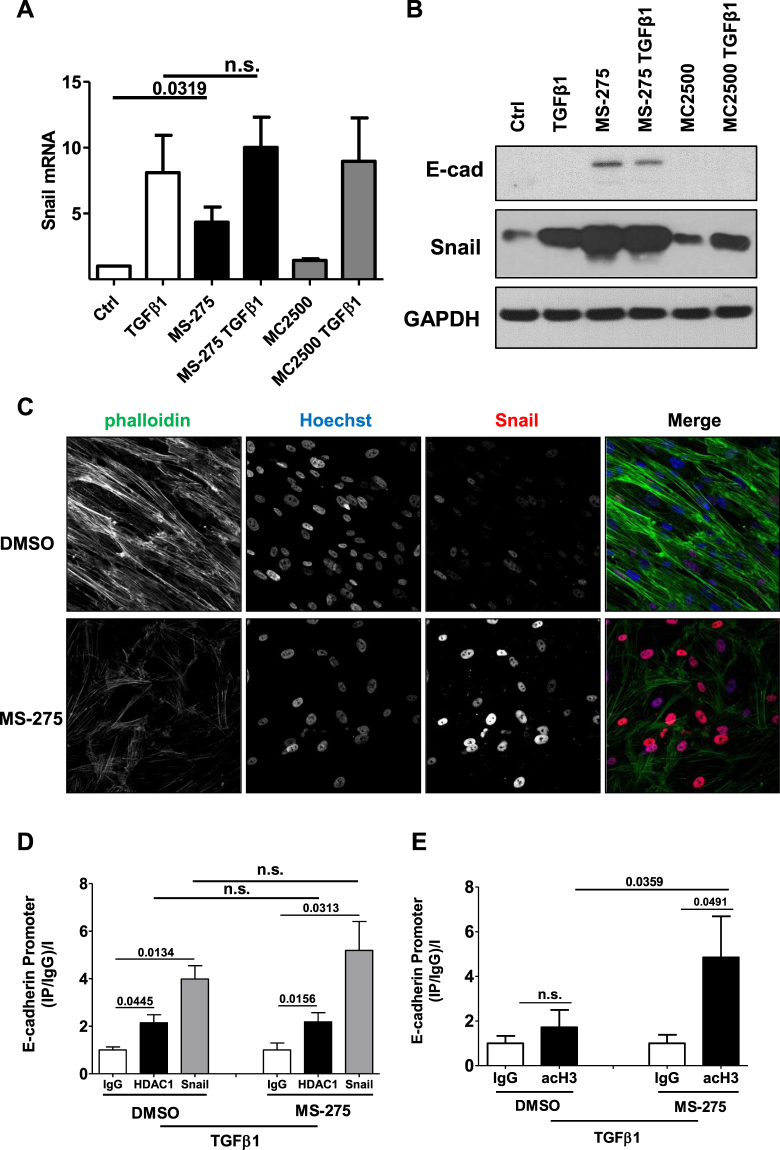
Treatment with MS-275 increases Snail expression while impairing its activity. (**A**) Effect of treatment with HDAC inhibitors over Snail expression in MCs. MCs were treated with DMSO vehicle (NT), with MS-275 (250 nM) or MC-2500 (250 nM) for 72 h. Samples were left untreated or were treated with TGFβ1 (2 ng/ml) for the last 24 h. Expression of Snail was evaluated on total RNA by qRT-PCR. n = 4. (**B**) Representative Western blot experiment of 5 performed showing expression of E-cadherin and Snail from cell lysates of MCs treated as above. GAPDH was used as a loading control. (**C**) Effect of MS-275 treatment on Snail expression and localization. MCs were treated with MS-275 (250 nM), or DMSO for 48 hr and then stimulated with TGFβ1 (2 ng/ml) for 24 h before processing for immunofluorescence with a monoclonal antibody against Snail. Cell nuclei are shown in blue (Hoechst 33342). Confocal images are shown from one representative experiment of three performed. (**D**) qPCR analysis of ChIP assays with anti-Snail (Snail) and anti-HDAC1 (HDAC1) antibodies and, as control, normal rabbit IgG (IgG) on chromatin from MeT5A cells treated with MS-275 for 72 h or with DMSO, and treated with TGFβ1 for 24 h (TGFβ1). Data show the enrichment of Snail and HDAC1 on Snail consensus binding site of human E-cadherin promoter. Values derived from 3 independent experiments are reported as means ± SEM and expressed as ((IP/IgG)/Input). Statistically significant differences are reported. **(E**) qPCR analysis of ChIP assays with anti-acH3 antibody and, as controls, normal rabbit IgG on chromatin from MeT5A cells treated with MS-275 for 72 h or left untreated (NT) and treated with TGFβ1 for 24 h (TGFβ1) when indicated. Data show the enrichment of H3 acetylation on Snail consensus binding sites of human E-cadherin promoter. Values derived from five independent experiments are reported as means ± SEM. and expressed as ((IP-IgG) %Input). Statistically significant differences are reported. P < 0.05 was considered significant.

When looking at the molecular mechanisms controlling MET, we first focused on Snail, the EMT master gene and direct repressor of E-cadherin expression. Unexpectedly, Snail expression was found markedly increased upon exposure to MS-275 both at mRNA and at protein level (Fig. 6A,B). These results were confirmed in MeT5A, a mesothelial cell line, where increased levels of both E-cadherin and Snail expression upon treatment with MS-275 were found (Suppl. Fig. 5A,B). IF experiments also showed increased nuclear staining of Snail in MCs having undergone a reversal towards a cobblestone phenotype after treatment with MS-275 (Fig. 6C).

The contradictory evidence of a *bona fide* MMT reversal in the presence of increased levels of the EMT-inducer Snail, suggests a MS-275-mediated Snail functional impairment.

We wondered whether Snail and HDAC1 binding to E-cadherin promoter was inhibited by the treatment with MS-275. We performed HDAC1 and Snail immunoprecipitation followed by RT-PCR (ChIP) on E-cadherin promoter in MeT5A cells in the presence of TGFβ1 to induce Snail expression.

**Figure 7:**
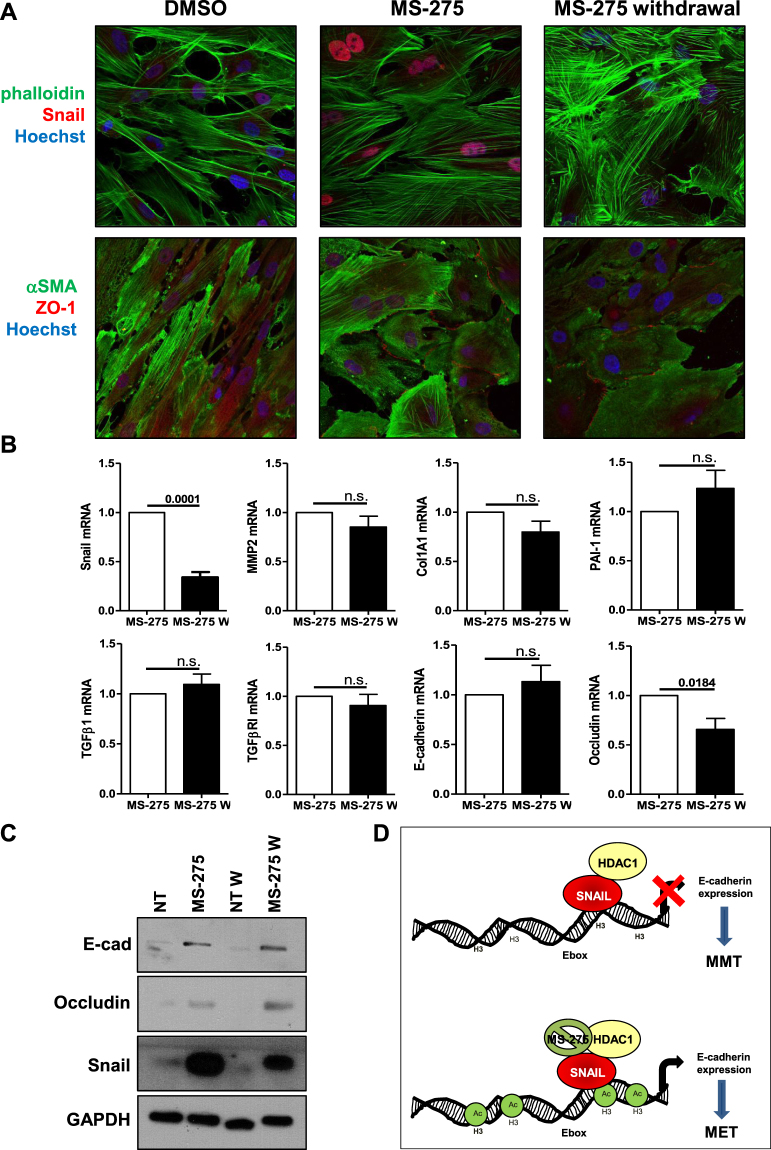
MS-275 withdrawal downregulates Snail expression, while maintaining an epithelial-like phenotype. (**A**) Effect of MS-275 withdrawal on MCs morphology and on Snail, αSMA and ZO-1 expression and localization. MCs were treated with MS-275 (250 nM) for 72 h. Then, the pharmacological inhibitor was washed out and the cells were cultured for three more days. MCs treated with MS-275 for three days were used as control. Cells were then processed for immunofluorescence using phalloidin, a monoclonal antibody against Snail, a monoclonal antibody against α-SMA and a polyclonal antibody against ZO-1. Cell nuclei are shown in blue (Hoechst 33342). Confocal images are shown from one representative experiment of three performed. (**B**) Effect of MS-275 withdrawal on epithelial and mesenchymal markers. Quantitative RT-PCR was performed on total RNA from MCs treated as above. Expression of Snail, MMP2, Col1A1, PAI-1, TGFβ1, TGFβRI, E-cadherin, Occludin was evaluated on total RNA by qRT-PCR. Data are reported as ratios of MS-275-treated and MS-275/withdrawal samples with respect to the non-treated samples. Bars represent means ± SEM of 5 experiments. (**C**) Western blots showing the expression of E-cadherin, Occludin and Snail in total cell lysates of MCs treated as above. GAPDH expression was used as a loading control. Representative experiment of 5 performed. (**D**) Proposed model describing the effect of MS-275 on Snail activity. Snail functional repressive activity (inhibition of E-cadherin expression) is HDAC1-dependent and thus is hampered by treatment with MS-275. P < 0.05 was considered significant.

As shown by Fig. 6D, in the presence of MS-275 neither Snail nor HDAC1 binding to E-cadherin promoter was impaired. Next, we verified histone H3 acetylation status on E-box within E-cadherin promoter upon treatment with MS-275. By performing acetylated histone H3 immunoprecipitation followed by RT-PCR on E-cadherin promoter, we found that histone H3 acetylation on E-cadherin promoter, reduced by TGFβ1, was restored in the presence of MS-275 (Fig. 6E). These results indicate that, despite increased expression of Snail and maintenance of Snail-HDAC1 binding to E-cadherin promoter, Snail functional repressive activity is HDAC1-dependent and thus is hampered by treatment with MS-275. Last, to evaluate the persistence of the epithelial-like phenotype obtained after treatment with MS-275, expression of Snail and of epithelial/mesenchymal markers were evaluated three days after withdrawal of MS-275. While expression of Snail was markedly reduced, the cobblestone-like morphology and ZO-1 membrane localization was maintained (Fig. 7A and Suppl. Fig. 6). Also, the expression of epithelial/mesenchymal markers was generally maintained after withdrawal of MS-275 (Fig. 7B,C). These results suggest that the treatment with MS-275 promotes the repositioning of MCs towards a new stable phenotype with epithelial-like features.

Overall, we demonstrated that inhibition of HDAC1 by MS-275 is sufficient to promote MMT reversal and inhibition of cellular invasiveness of MCs from effluent of PD patients having undergone MMT *in vivo* and in a model of PD fluid exposure *in vitro*. Moreover, treatment with MS-275 hampers Snail activity through altered histone H3 acetylation status on specific promoters (Fig. 7D).

## Discussion

Aim of this study was to evaluate the effect of HDACs in the re-acquisition of epithelial-like features in MCs isolated from effluent of PD patients and to elucidate mechanisms involved. The rationale for the use of HDAC pharmacological inhibitors was provided by our finding that epithelial-like MCs showed higher H3 acetylation on E-cadherin promoter with respect to mesenchymal-like cells.

When analyzing different HDAC inhibitors, we found that while the effect of TSA (pan-HDAC inhibitor) and MC1568 (HDAC4-6-8 inhibitor) were limited to a rescue of E-cadherin expression, treatment with MS-275 (HDAC1-3 inhibitor) was sufficient to promote a *bona fide* MMT reversal characterized by increase of epithelial markers, decrease of mesenchymal markers, conformational change, reduction of directed migration (scratch assay) and total inhibition of three dimensional invasiveness through Matrigel. Due to their mesodermal origin, MCs have in basal conditions an epithelial-like morphology co-expressing epithelial and mesenchymal markers, such as E-cadherin, cytokeratins, vimentin and desmin^20^. Once stimulated by TGFβ1 often in combination with TLR ligands, MCs undergo MMT and may became indistinguishable from myofibroblasts obtained from other sources^26^.

The ability to stably acquire mesenchymal features makes these cells a privileged experimental model for the study of mechanisms controlling EMT dynamics and especially the role of epigenetics. In ‘truly epithelial cells’ such as hepatocytes, TGFβ1 withdrawal is sufficient to determine recovery of E-cadherin levels and to induce Snail downregulation^27^. In MCs, induction of MMT leads to a stable acquisition of a ‘mesenchymal-like’ phenotype, characterized by reduced or absent E-cadherin and constitutively elevated Snail expression levels^11,20,28^.

To our knowledge, our study first deals on the effect of HDAC1 inhibition on MMT of MCs. A recent report analyzed the role of a selective HDAC6 inhibitor, tubastatin, in the inhibition of peritoneal MMT and fibrosis^29^. Interestingly, in apparent contradiction to our results, treatment with the inhibitor of histone acetyltransferase C646 was reported to limit the MMT induced by glucose in a MC line^30^.

The specificity of MS-275 was warranted by the use of its *meta* isomer MC2500, which was ineffective. MS-275 is an inhibitor of HDAC1 and to lesser extent, of HDAC2 and HDAC3 activity^31^. At the concentration used in this study (250 nM), this pharmacologic inhibitor is specific for the HDAC1 isoform. Genetic silencing experiments confirmed that HDAC1 plays a major role in MMT reversal observed in MCs. As a further confirmation of cellular activity, MS-275 inhibited MMT induced by treatment of epithelial-like MCs with PD fluid *in vitro*.

The role of MS-275 in EMT has been evaluated in other experimental systems, especially in tumors. MS-275 at higher concentrations was demonstrated to reverse EMT in ERa-negative breast cancer cell lines^17^. In a non-tumoral experimental setting, MS-275 was demonstrated to limit renal fibrosis induced by renal fibroblasts activation, although no information was provided about renal epithelial cells^32^.

As previously mentioned, TSA and MC1568 effects are limited to a rescue of E-cadherin expression. No data are available in the literature about the effect of MC1568 in E-cadherin expression and EMT. Since MC1568 at the used dose (10 µM) inhibits HDAC6 and HDAC8, conceivably inhibition of other HDACs is needed to obtain a more general reprogramming towards the epithelial-like phenotype in MCs^22,23^. With regard to TSA, there is an apparent conflict with other studies performed generally in epithelial cell lines or tumors where TSA treatment inhibited EMT^33,34^. However, in these studies TSA was used at toxic concentrations for primary MCs (data not shown).

Besides epithelial/mesenchymal marker expression and cell morphology, we focused on the regulation of cellular functions. Since cells coexpressing epithelial cytokeratin and a mesenchymal marker (α-SMA or fsp1) have been found in the submesothelial stroma of fibrotic peritoneum, it has been hypothesized that MCs invasion and subsequent production of ECM proteins, VEGF and inflammatory/chemotactic factors is a main mechanism of fibrosis^9,35^. Alternatively, it has been proposed that MCs undergoing a partial EMT and remaining at the mesothelial layer would participate to the EMT process secreting proinflammatory/pro-fibrotic factors^36^. Studies of lineage tracing demonstrated that MCs may invade the sub-mesothelial stroma, although the number of MCs generating myofibroblasts and the relevance of this process may change in different models of peritoneal fibrosis^37,38^. Our data demonstrate that, while the maintenance of a reduced horizontal mobility may allow devoid areas of peritoneum to be repopulated even in the presence of HDAC inhibitors allowing re-peritonealization^39,40^, potentially harmful invasion of the sub-mesothelial stroma is totally blocked.

Previous studies focused on mechanisms maintaining a mesenchymal-like phenotype in MCs from PD patients. Due to the relevance of TGFβ signaling, SMAD dependent and independent mechanisms were elucidated^3,41^. Constitutive hyper-activation of MEK-ERK1/2-Snail and TAK1/NF-κB pathways was described in these cells and pharmacological blockage induced a partial MMT reversal^11,28^.

Exogenous administration of BMP7, an antagonist of TGFβ1, was sufficient to induce MMT reversal^12,42^. MCs exposed to pro-inflammatory stimuli secrete large amounts of TGFβ1, whose production may induce signaling loops playing a role in the maintenance of a mesenchymal-like phenotype^43^.

To this extent, our studies complement these previous discoveries, since we demonstrate that sensitivity of MCs to TGFβ1 is blocked by treatment with an HDAC1-3 specific inhibitor, MS-275. The lack of cellular response to TGFβ1 observed upon treatment with MS-275 may arise by: (i) reduction of endogenous TGFβ1 expression; (ii) reduced levels of TGFβRI; (iii) inhibition of downstream mediators, such as Snail.

When focusing on Snail expression, we unexpectedly found that Snail levels were increased upon treatment with MS-275. Since direct targets, such as E-cadherin and Occludin, were increased as well, we hypothesized that Snail activity would be hampered despite increased expression.

Peinado *et al*. demonstrated that E-cadherin repression is mediated by Snail through the recruitment of the Sin3A/HDAC1/HDAC2 complex^44^. The recruitment to Snail binding site of other pivotal epigenetic factors including Polycomb Repressive Complex 2 (PRC2) have also recently been demonstrated to be crucial for Snail activity^45^.

We observed that treatment with MS-275, while non influencing Snail/HDAC1 recruitment on E-cadherin promoter, did not result in the expected local H3 histone deacetylation, thus causing the impairment of the TGFβ1-induced Snail-mediated repression (Fig. 7D).

Interestingly, this inhibition of Snail activity is inhibitor-specific or dependent on cellular context, since other HDAC inhibitors such as valproic acid, sodium butyrate and SAHA have been demonstrated to promote EMT through upregulation of Snail in epithelial tumors^15,16^.

Other mechanisms proposed for the effect of MS-275 in EMT were reduction of SMAD3, EGFR, STAT3 phosphorylation^32^. Our proposed mechanism, focusing on the effect of increased H3 histone acetylation on E-cadherin promoter, explains the observed functional inactivation of the TGFβ1/Snail axis. However, we do not exclude that other mechanisms such as those above cited, may play a role in HDAC1 inhibition especially *in vivo*.

Since HDAC inhibitors are already used in cancer therapy and are on trials for other non tumoral/fibrotic diseases, the understanding of the role of HDAC1 in the maintenance of a mesenchymal state in MCs has both a basic and a translational relevance. These discoveries may have also a broader impact in relevant clinical issues unrestricted to PD practice, such as in the prevention of post-operative peritoneal adhesions, and in the control of peritoneal metastases.

## Methods

### Cells

Effluent-derived MCs were isolated from 17 clinically stable PD patients as described previously^10^. Baseline clinical data from these patients are reported in Table 1. MCs from PD effluents express the standard mesothelial markers intercellular adhesion molecule (ICAM)-1 and cytokeratins 8–18, although at lower levels than healthy HPMCs. MC cultures are negative for the endothelial marker CD31 and the pan-leukocyte marker CD45 ^6,11,28^. Effluent-derived MCs were cultured in Earle’s M199 supplemented with 10% FBS (GIBCO® Life Technology, Monza, Italy) and antibiotics.

**Table 1:** Baseline clinical data of patients enrolled in this study.

PATIENTS	Sex	Age	Cause of kidney failure	Diabetes	Hypertension	Years from starting PD	PD tecnique	Exchanges	Glucose (MG/DL)	Peritonitis	Hemoperitoneum	Escapes
1	M	71	Glomerulonephritis	NO	YES	3	CAPD	2	2996	0	NO	YES
2	M	66	Glomerulonephritis	NO	YES	5	CAPD	2	4540	0	NO	NO
3	M	56	Glomerulonephritis	NO	YES	4	CAPD	4	9080	2	NO	YES
4	M	84	Chronic Pielonephritis	NO	YES	6	CAPD	1	2270	1	NO	NO
5	F	70	Diabetes Hypertension	YES	YES	3	CAPD	4	9080	0	NO	NO
6	M	74	IGA Glomerulonephritis	NO	YES	5	CAPD	2	3733	0	NO	YES
7	M	70	Diabetes Hypertension	YES	YES	5	CAPD	2	4540	2	NO	YES
8	M	70	IGA Glomerulonephritis	NO	YES	3	CAPD	2	3768	0	NO	NO
9	M	61	Chronic Pielonephritis	NO	YES	2	CAPD	1	1498	0	NO	NO
10	M	67	Chronic Pielonephritis	NO	YES	1	CAPD	2	4540	1	NO	YES
11	F	65	Diabetes	YES	YES	1	CAPD	2	3768	0	NO	NO
12	F	82	Glomerulonephritis	NO	YES	0	CAPD start	1	2270	0	NO	NO
13	M	47	Membranous GN	NO	YES	3	APD		17143	1	NO	NO
14	M	40	ADPKD	NO	YES	0	CAPD start	1	1498	0	NO	NO
15	M	60	IGA Glomerulonephritis	NO	YES	0	CAPD start	1	1498	1	NO	NO
16	M	62	IGA Glomerulonephritis	NO	YES	0	CAPD start	1	1498	0	NO	NO
17	M	64	Diabetes Hypertension	YES	YES	0	CAPD start	1	1498	0	NO	NO

To enhance EMT-like features, MCs were treated with TGFβ1 (2 ng/ml) as described previously^19,20^. The cytokine dose used is in the range of those detected in peritoneal-dialysis fluids from patients with peritonitis^46^ and are similar to those used in previous studies^20,47^. In some experiments, epithelial-like MCs were treated for four days with stay safe balance 4.25% (glucose 4.25%) PD fluid (used at a concentration of 1:1 with complete 199 Medium). The next three days, cells were maintained with PD fluid in the presence of DMSO or of MS-275 (250 nM).

The study was performed according to guidelines from the ethics committee of Sant’Andrea Hospital, Sapienza University (Rome, Italy). Written informed consent was obtained from all PD patients. The protocol and informed consent were reviewed and approved by the Ethics Committee of Clinic Investigation of Sapienza University ref: 4697_2017 (Roma, Italy). The human mesothelial cell line MeT5A (ATCC, Rockville, MD) was cultured in Earle’s M199 as above.

### Antibodies and chemicals

Monoclonal antibodies against Snail and HDAC1 (for WB experiments) were from Cell Signaling technology; polyclonal antibodies against Snail (H-130, for ChIP experiments) were from Santa Cruz Biotech (Dallas, TX); monoclonal antibodies against E-cadherin were from BD (Becton-Dickinson Laboratories, Mountain View, CA); monoclonal antibodies against α-SMA were from Sigma (Saint Louis, MO); polyclonal anti-ZO-1 were from Zymed (Invitrogen, Carlsbad, CA); polyclonal antibodies against HDAC1 (for ChIP experiments), GAPDH, Actin, acetyl-H3 and rabbit IgG control were from Millipore (Merck, Kenilworth, NJ). Matrigel Matrix Growth Factor Reduced (GFR) was from BD bioscences (Milan, Italy). Magnetic beads (Dynabeads) were from Invitrogen (Carlsbad, CA).

Rhodamine-phalloidin and Hoechst 33342 were from Invitrogen. TSA was from Selleck (Houston, TX), MC1568, MS-275, MC2500 were from Mai lab. Stay safe balance 4.25% PD fluid was from Fresenius (Bad Homburg, Germany).

### Western blotting

Monolayers MCs or MeT5A cells were lysed in modified RIPA buffer containing: 50 mM Tris-HCl, pH 7.4; 1% NP-40; 0.1% SDS; 0.25% Nadeoxycholate; 150 mM NaCl; 1 mM EDTA; 1 mM EGTA; 1 mM PMSF; 1 μg/ml each of aprotinin, leupeptin and pepstatin; and 25 mM NaF (all from Sigma). Equal amounts of protein were resolved by SDS-PAGE. Proteins were transferred to nitrocellulose membranes (Biorad, Hercules, CA) and probed with antibodies using standard procedures. Nitrocellulose-bound antibodies were detected by chemiluminescence with ECL (Cyanagen, Bologna, Italy).

### Reverse-transcriptase polymerase chain reaction

RNA, extracted from cell cultures with ReliaPrep™ RNA Tissue Miniprep System (Promega, Madison, WI, USA), was reverse transcribed with iScriptTM c-DNA Synthesis Kit (Bio-Rad Laboratories, Inc., Hercules, CA, USA) according to the manifacturer’s instructions. cDNAs were amplified by qPCR reaction using GoTaq® qPCR Master Mix (Promega, Madison, WI, USA). The following specific primer pairs were used**:** for L34: 5′GTCCCGAACCCCTGGTAATAG3′ and 5′GGCCCTGCTGACATGTTTCTT3′; for MMP2: 5′ATGCCGCCTTTAACTGGAG3′ and 5′GGAAAGCCAGGATCCATTTT3′; for Col1A1: 5′AGCCAGCAGATCGAGAACAT3′ and 5′TCTTGTCCTTGGGGTTCTTG3′; for Occludin: 5′AAGGTCAAAGAGAACAGAGCAAGA3′ and 5′TATTCCCTGATCCAGTCCTCCTC3′; for Snail: 5′CACTATGCCGCGCTCTTTC3′ and 5′GCTGGAAGGTAAACTCTGGATTAGA3′; for PAI-1: 5′AGTGGACTTTTCAGAGGTGGA3′ and 5′GCCGTTGAAGTAGAGGGCATT3′; for TGFβ1: 5′AAGGACCTCGGCTGGAAGTG3′ and 5′CCCGGGTTATGCTGGTTGTA3′; for E-cadherin: 5′TACGCCTGGGACTCCACCTA3′ and 5′CCAGAAACGGAGGCCTGAT3′; for TGFβRI: 5′AACTTCCAACTACTGGCCCT3′ and 5′GGTGAATGACAGTGCGGTTG3′ for HDAC1: 5′CATCGCTGTGAATTGGGCTG3′ and 5′CCCTCTGGTGATACTTTAGCAGT3′. Relative amounts, obtained with 2^(−ΔCt)^ method, were normalized with respect to the housekeeping gene L34. Statistical significance was determined with a t-test with GraphPad Prism version 5.0 (La Jolla, CA, USA). Differences were considered significant at P < 0.05. Values are reported in the graphs.

### Chromatin immunoprecipitation assay (ChIP)

ChIP analysis was performed as reported previously^45^. 5 μg of primary antibody or rabbit IgG were used. After washes, samples were eluted with the elution buffer (for acethyl-H3, TE 1x, sodium dodecyl sulfate (SDS) 0.5%; for Snail and HDAC1, NaHCO_3_ 100 mM, SDS 1%), treated with RNase A and with proteinase K (Sigma). The extracted DNA was used in the qPCR analyses. The following specific primer pairs were used: for E-cadherin promoter EBOX, 5′GGTGAACCCTCAGCCAATCA3′ and 5′CACAGGTGCTTTGCAGTTCC3′; for GAPDH promoter (used as a positive control for determining H3 acetylated IP efficiency), 5′TACTAGCGGTTTTACGGGCG3′ and 5′TCGAACAGGAGGAGCAGAGAGCGA3′. Data were expressed as (IP-IgG)/Input.

### siRNA-mediated HDAC1 knockdown

100 × 10^3^ MCs were seeded on 12-well plates 24 h prior transfection. Cells were transfected with either 40 pmol of siRNA against human HDAC1 (5′CAGCGACUGUUUGAGAACC3′ or the same amount of siRNA against GFP (5′-GGCU ACGUCCAGGAGCGCACC-3′) from MWG biotech (Ebersberg, Germany) and 2 μl Lipofectamine® RNAiMAX Reagent from Thermo Fisher Scientific (Waltham, MA USA) in 200 μl Optimem from Gibco (Waltham, MA USA). 1 ml of supplemented medium per well was also added. 72 h after transfection, knockdown efficiency was determined by RT-PCR and western blot.

### Confocal microscopy and immunofluorescence

MCs were fixed for 20 minutes in 3% formaldehyde in PBS, permeabilized in 0.2% Triton X-100/PBS for 5 minutes, and blocked with 2% BSA for 20 minutes. Secondary antibodies (conjugated to Alexa-647, -488 and -541) were from Pierce Chemical Company (Rockford, IL). Confocal images were acquired using a Leica SP5 spectral confocal microscope. The spectral technology allows discrimination between yellow and green fluorescence.

### Scratch assay

MCs were allowed to reach 100% confluency. Cells were pretreated with DMSO, 250 nM MS-275 or MC2500 at the same concentration for 48 h in culture medium until reaching 100% confluency. A scratch wound was created using the culture-insert 2 wells in µ-Dish from ibidi (Martinsried, Germany). Micrographs were taken at time 0 and 18 h after the scratch. Three independent experiments were performed. For microscopy time lapse experiment, after insert removing the wound closure was monitored for 15 h acquiring bright-field images every 30 minutes. Images were acquired at the Olympus iX83 FluoView1200 laser scanning confocal microscope using an UPLSAPO10x2, NA 0.40 air objective. Images ware stitched using Olympus FluoView software.

### 3-dimensional Invasion Assay

3‐dimensional invasion assays were performed as in^35^, MCs (1.5 × 10^5^) were treated with 250 nM MS-275, 250 nM MC2500 or DMSO for 12 h and then seeded in triplicate in ibidi 15‐well slides (µ-Slide Angiogenesis) and allowed to attach for 3 h. 40% Matrigel (40 μl) in serum‐free medium was laid over the cells. After 1 h, 50 μl full medium containing 20% serum (and MS-275or vehicle) was added and cells incubated for 72 h. Cells were fixed with 4% paraformaldehyde (PFA), permeabilized with 0.25% Triton PBS, and stained for 12 h with rhodamine‐phalloidin (to stain F-actin) and Hoechst (to stain nuclei) in PBS. After washes, cell were mounted using the ibidi mounting medium. Confocal images were acquired at the Olympus iX83 FluoView1200 laser scanning confocal microscope using an UPLSAPO10x2, NA 0.40 air objective. Each stack consists of individual images with a z‐step of 5 μm. The 3D rendering was performed using the Imaris image analysis software v.8.1.2 (Bitplane). The invasion quantification was performed with the software Huygens Professional v.17.04 (Scientific Volume Imaging, The Netherlands). Three independent experiments were performed.

### Statistical analysis

Statistical significance was determined with a *t*-test using GraphPad Prism version 5.0 (La Jolla, CA, USA). Differences were considered significant at *P* < 0.05.

## Electronic supplementary material

Supplementary figures
