# Supplementary material for: HDAC1 inhibition by MS-275 in mesothelial cells limits cellular invasion and promotes MMT reversal
Source: Sci Rep. 2018 May 31;8:8492. doi: 10.1038/s41598-018-26319-2 (PMC5981641; doi:10.1038/s41598-018-26319-2)
Supplement: Supplementary file 1 — Supplementary figures [file 41598_2018_26319_MOESM1_ESM.docx]

#### **HDAC1 inhibition by MS-275 in mesothelial cells limits cellular invasion and promotes MMT reversal**

Lucia Rossi^1,6^, Cecilia Battistelli^1,6^, Valeria de Turris^2^, Valeria Noce^1^, Clemens Zwergel^3^, Sergio Valente^3^, Alessandra Moioli^4^, Andrea Manzione^4^, Marco Palladino^4^, Veronica Bordoni^5^, Alessandro Domenici^4^, Paolo Menè^4^, Antonello Mai^3^, Marco Tripodi^*1,5^, Raffaele Strippoli^*1,5^.

*^1^Department of Cellular Biotechnologies and Hematology, Section of Molecular Genetics, Sapienza University of Rome, Rome, Italy*

*^2^ Center for Life Nano Science@Sapienza, Istituto Italiano di Tecnologia, Rome, Italy*

*^3^ Dipartimento di Chimica e Tecnologie del Farmaco "Sapienza" Università di Roma, Rome, Italy*

*^4^Department of Clinical and Molecular Medicine, Sapienza University of Rome, Nephrology Unit, Sant'Andrea University Hospital, Rome, Italy*

*^5^Gene Expression Laboratory, National Institute for Infectious Diseases "Lazzaro Spallanzani" I.R.C.C.S., Rome, Italy.*

*^6^These authors contributed equally*

^*^Authors for correspondence:

Raffaele Strippoli MD, PhD

Assistant Professor, Department of Biotechnologies and Haematology

Sapienza University, Rome, Italy

Tel.[+390649918236](tel:+39%2006%204991%208236)

Fax [+390649918251](tel:+39%2006%204991%208251)

[raffaele.strippoli@uniroma1.it](mailto:raffaele.strippoli@uniroma1.it)

Marco Tripodi, PhD

Full Professor, Director of the Department of Biotechnologies and Haematology,

Sapienza University, Rome, Italy

Tel.+390649918244

Fax +390644252865

tripodi@bce.uniroma1.it

**
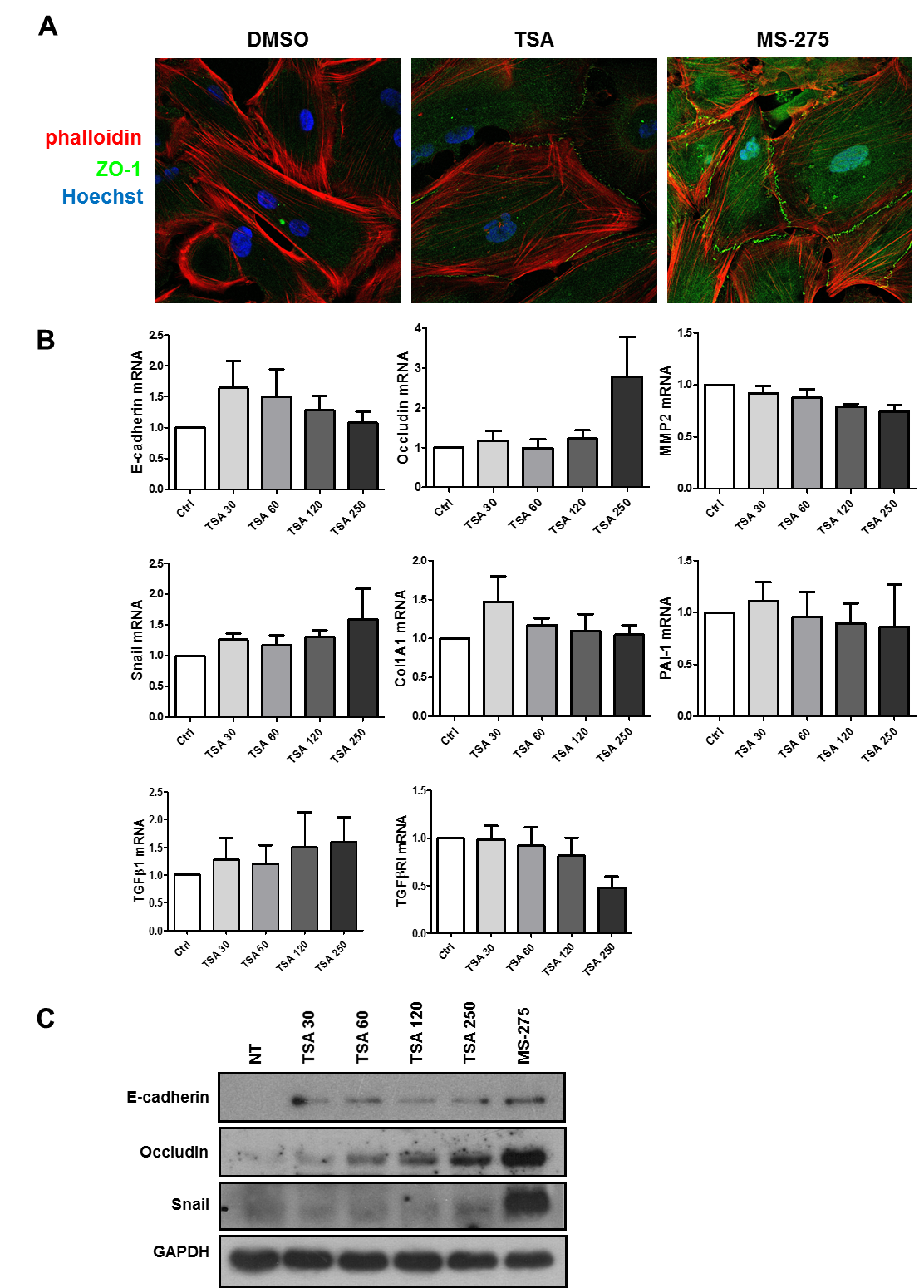
Suppl. Fig. 1** Effect of TSA in MMT reversal. (**A**) MCs were treated with DMSO vehicle (Ctrl) or with TSA (TSA) (250 nM) or MS-275 (250 nM) for three days. Cells were fixed, permeabilized and stained with phalloidin and with a polyclonal antibody against ZO-1. Images were acquired by confocal microscopy. Cell nuclei are shown in blue (Hoechst 33342). Confocal images are shown from one representative experiment of three performed. (**B**) Quantitative RT-PCR was performed on total RNA from MCs treated for three days with TSA (30-250 nM) at the concentrations shown in the figure. Expression of E-cadherin, Occludin, MMP2, Snail, Col1A1, PAI-1, TGFβ1, TGFβRI, was evaluated on total RNA by qRT-PCR. Bars represent means ± SEM of 5 experiments (**C**) Western blots showing the expression of E-cadherin, Occludin and Snail in total cell lysates of MCs treated as above. GAPDH expression was used as a loading control. Representative experiment of 5 performed. P < 0.05 was considered significant.

**A**

**B**

**C**

**D**

**Suppl. Fig. 2**. Effect of MC2500 on mesenchymal markers expression in MCs derived from peritoneal effluent of PD patients. MCs were treated with DMSO vehicle (Ctrl), with MS-275 (250 nM) or with MC2500 (250 nM) for 72 h. Samples were left untreated or were treated with TGF-β1 (2 ng/ml) for the last 24 h. Expression of MMP2 (**A**), Col1A1 (**B**), TGFβ1 (**C**), TGFβRI (**D**), was evaluated on total RNA by qRT-PCR. Bars represent means ± SEM of 4 experiments. P < 0.05 was considered significant.

**ZO-1**


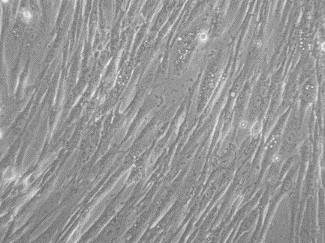

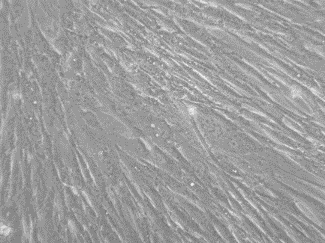

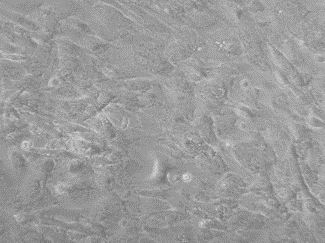

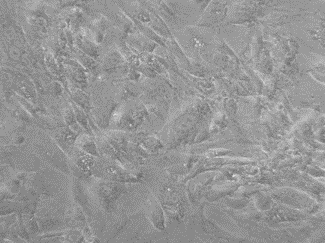

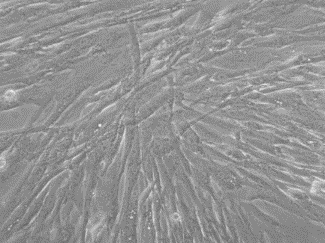

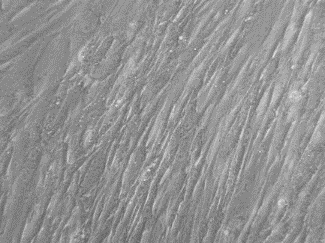


**TGFβ1**

**NT**

**MS-275**

**MC2500**

**DMSO**

**A**

**DMSO**


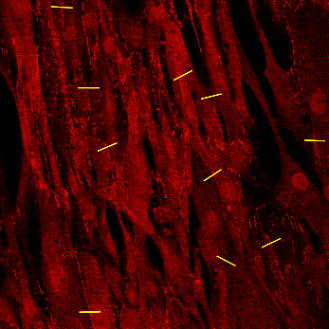

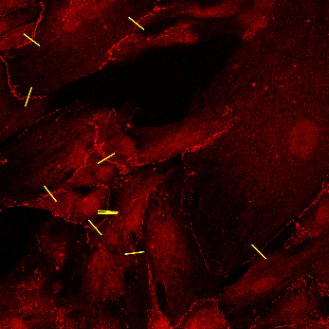

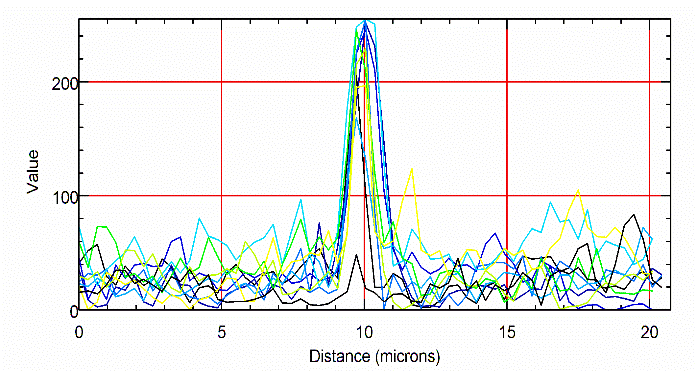

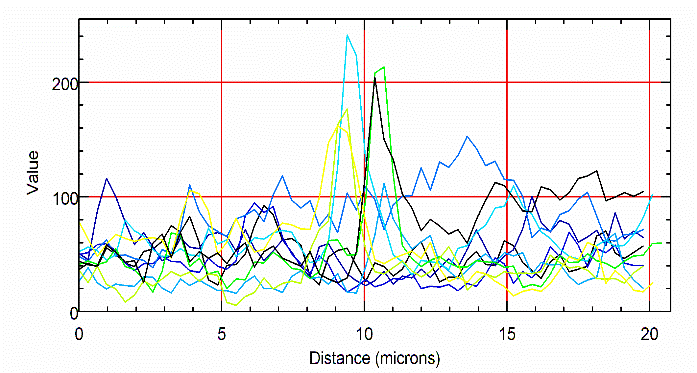


**MS-275**

**TGFβ1**

**NT**


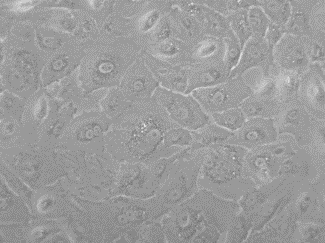

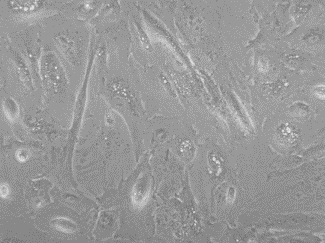

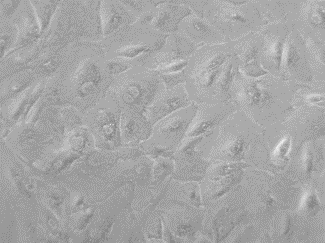

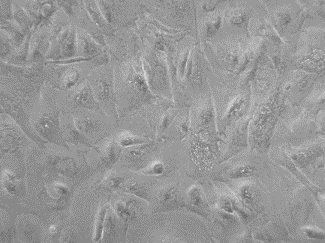

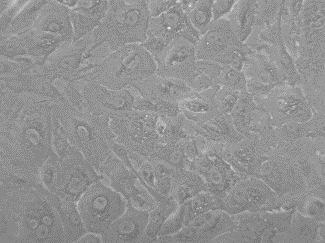

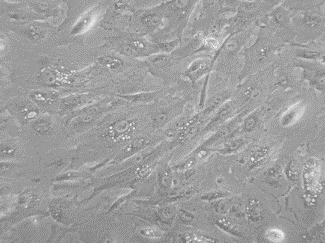


**B**

**C**

**Suppl. Fig. 3**. Effect of MS-275 on ZO-1 localization and cellular junctions and on the reacquisition of an epithelial-like morphology (**A)** Fluorescence intensity profiles from ZO-1 staining were analyzed with Leica LAS-AF software. In brief, lines were drawn from a few microns from the cell edge towards the cell inside and fluorescence intensity was obtained. 10 lines per condition from different cells were analyzed. Fluorescence intensity concentrates in the middle of the lines, corresponding to cell-to-cell junctions, in MCs treated with MS-275. (**B-C**) Bright field microscopy images are shown from the experiment illustrated in **Fig.** **4A-B**. Both untreated and TGFβ1 treated MCs from 2 different PD patients are shown.


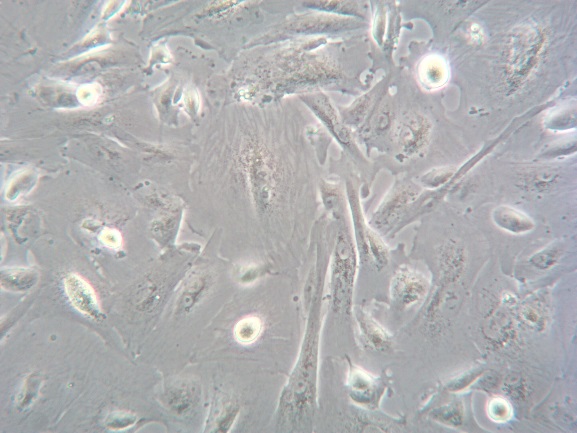

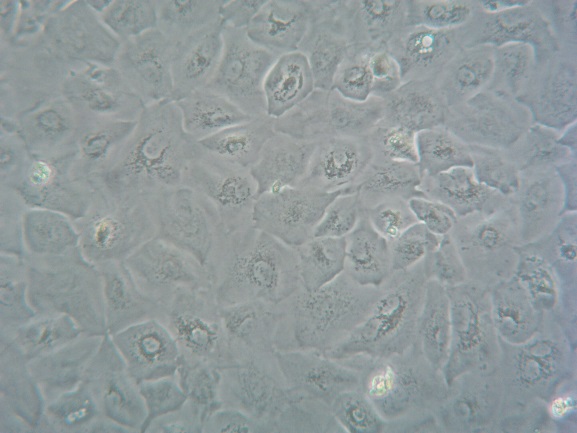

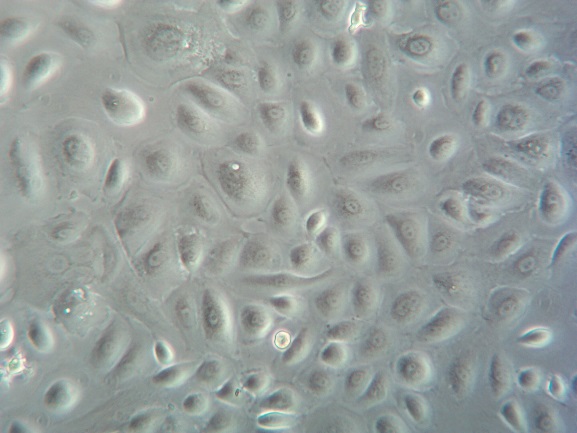


**DMSO**

**Dialysis Fluid**

**MS-275**

**Dialysis Fluid**

**MCs I MCs II**


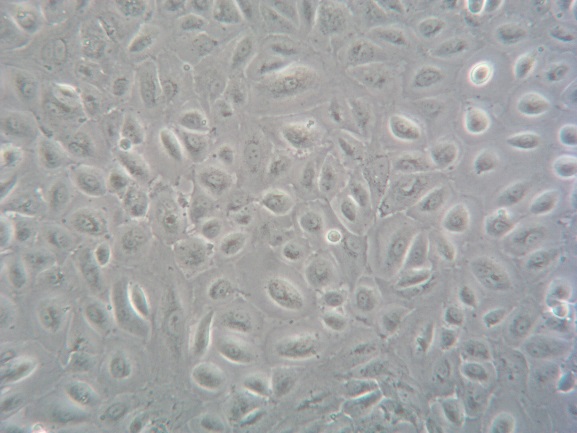

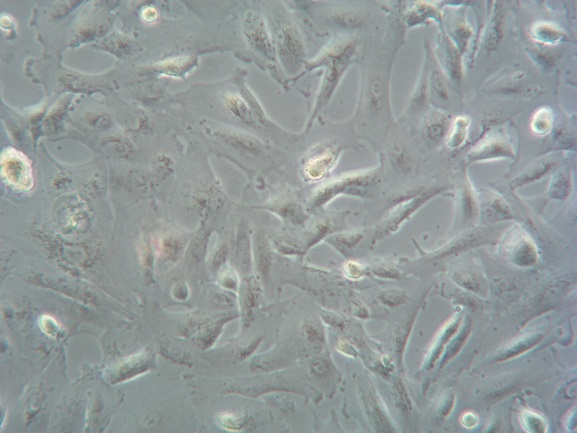

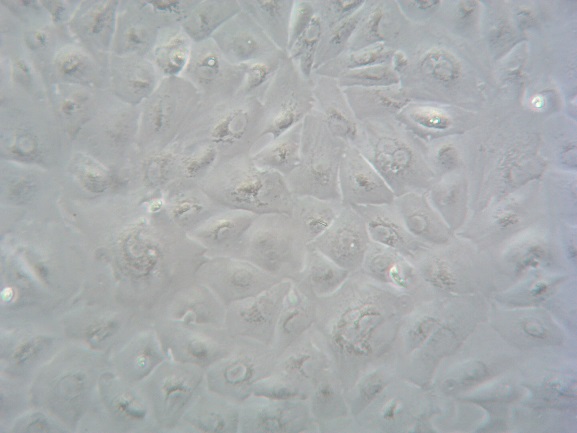


**Suppl. Fig. 4** Bright field microscopy images (two different MCs samples are shown) from the experiment illustrated in **Fig. 4C**.

**A**

**GAPDH**

**MC2500 TGFβ1**

**Snail**

**E-cad**

**NT**

**TGFβ1**

**MS-275**

**MS-275 TGFβ1**

**MC2500**


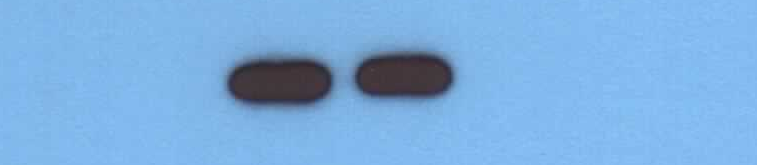

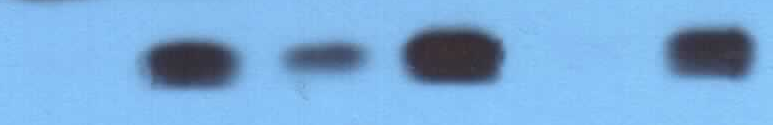

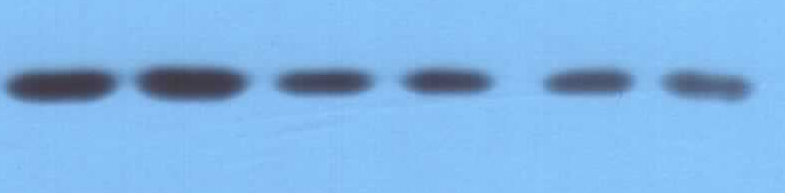


**B**


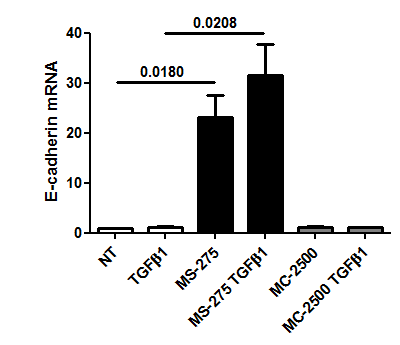

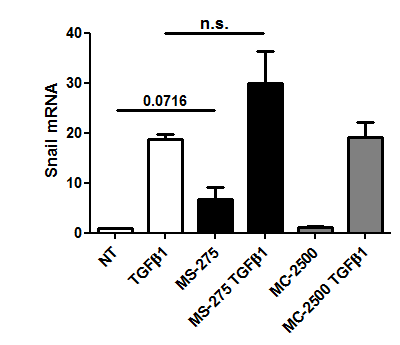


**Suppl. Fig. 5**: Effect of treatment with MS-275 on Snail and E-cadherin expression in MeT5A cells. MeT5A cells were treated with DMSO vehicle (NT), with MS-275 (250 nM) or MC2500 (250 nM) for 72 h. Samples were left untreated or were treated with TGF-β1 (2 ng/ml) for the last 24 h. (**A**): Expression of Ecadherin (top) and of Snail (bottom) was evaluated on total RNA by qRT-PCR and normalized respect to the human housekeeping gene L34 by using the 2^(-ΔCt method). Bars represent means ± SEM of 3 experiments. (**B**): Representative Western blot experiment from 3 performed showing expression of E-cadherin and Snail from cell lysates of MeT5A cells treated as above. GAPDH was used as a loading control.


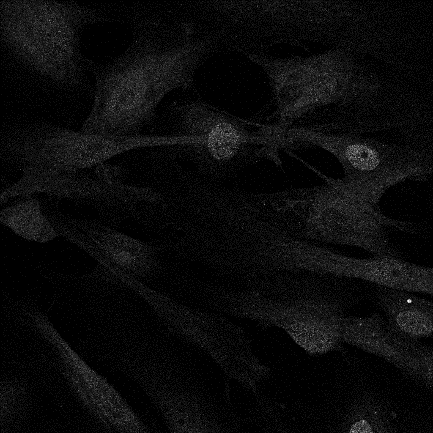

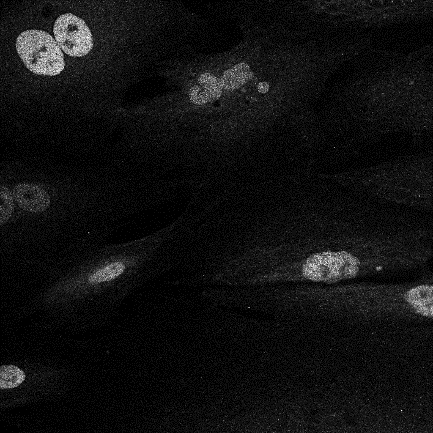

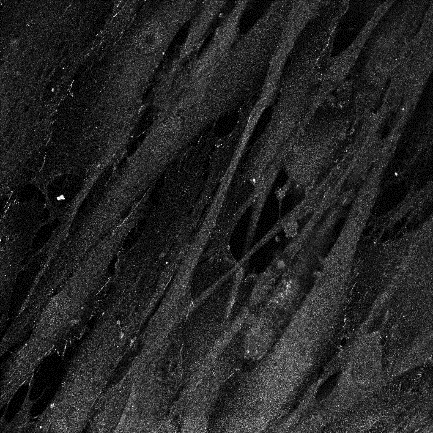

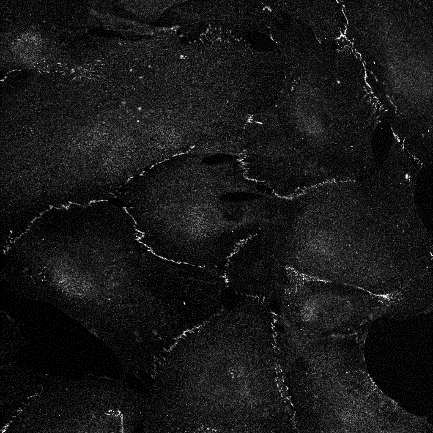

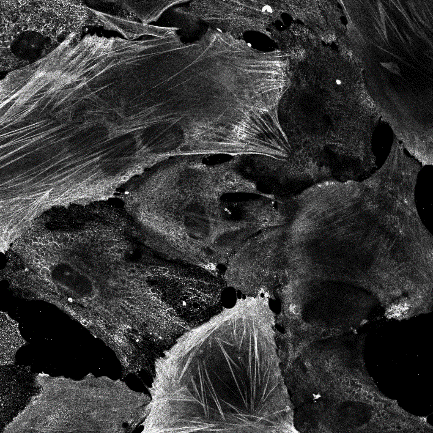

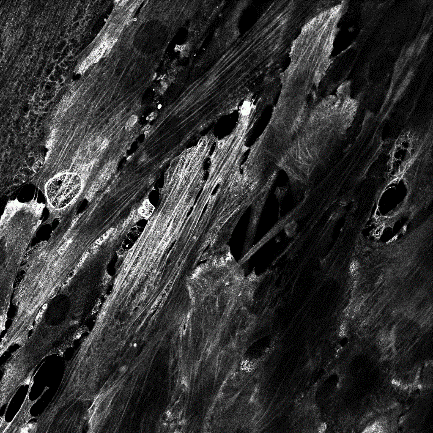

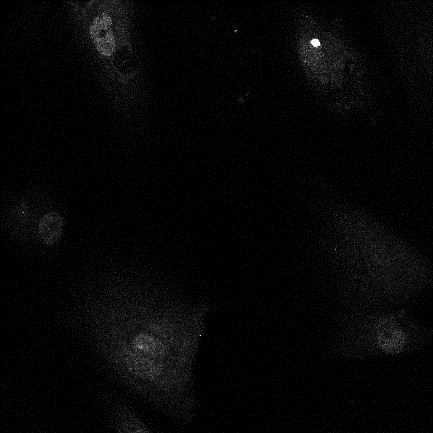

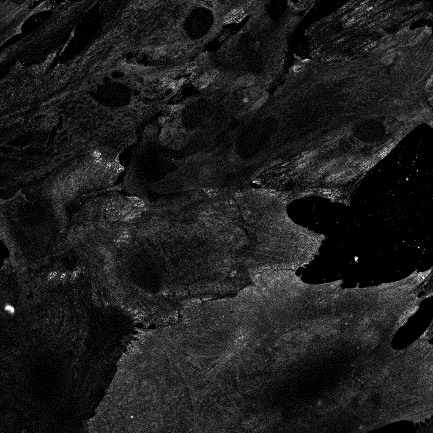

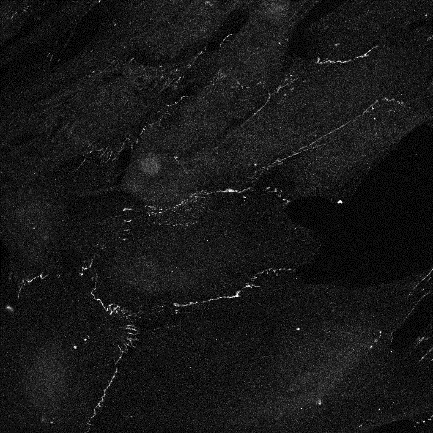


**MS-275**

**MS-275 withdrawal**

**NT**

**Snail**

**ZO-1**

**αSMA**

**Suppl. Fig. 6** Single fluorescence images shown in the **Figure 7A.**
